# The performance of plasma pTau181 and pTau217 in distinguishing Alzheimer's disease from various neurodegenerative disorders, psychiatric disorders, and cognitively unimpaired controls

**DOI:** 10.1177/13872877261431800

**Published:** 2026-03-23

**Authors:** Juho-Antti Rissanen, Sari Kärkkäinen, Kasper Katisko, Aleksi Vanninen, Antti J. Luikku, Tuomas Rauramaa, Tadeusz Musialowicz, Merja Kokki, Valtteri Julkunen, Anne M. Portaankorva, Annakaisa Haapasalo, Eino Solje, Päivi Hartikainen, Ville Leinonen, Tarja Kokkola, Sanna-Kaisa Herukka

**Affiliations:** 1 Institute of Clinical Medicine - Neurology, 4344 University of Eastern Finland, Kuopio, Finland; 2 Neuro Center - Neurology, 60650 Kuopio University Hospital, Kuopio, Finland; 3 Institute of Clinical Medicine - Neurosurgery, 4344 University of Eastern Finland, Kuopio, Finland; 4 Neuro Center - Neurosurgery, 60650 Kuopio University Hospital, Kuopio, Finland; 5 Institute of Clinical Medicine, Pathology, 4344 University of Eastern Finland, Kuopio, Finland; 6 Department of Pathology, 60650 Kuopio University Hospital, Kuopio, Finland; 7 Anaesthesiology and Intensive Care, 60650 Kuopio University Hospital, Kuopio, Finland; 8 School of Medicine, 4344 University of Eastern Finland, Kuopio Finland; 9 Clinical Neurosciences, Faculty of Medicine, 3835 University of Helsinki, Helsinki, Finland; 10 A.I. Virtanen Institute for Molecular Sciences, 4344 University of Eastern Finland, Kuopio, Finland

**Keywords:** Alzheimer’s disease, biomarkers, frontotemporal dementia, Lewy body disease, normal pressure hydrocephalus, psychiatry, synucleinopathies

## Abstract

**Background:**

Plasma phosphorylated tau isoforms 181 (pTau181) and 217 (pTau217) are promising Alzheimer's disease (AD) biomarkers.

**Objective:**

We evaluated the performance of pTau181 and pTau217 in the differential diagnostics between AD, other neurodegenerative diseases and non-neurodegenerative participants.

**Methods:**

We included 104 patients with neurodegenerative diseases (37 with AD, 21 with synucleinopathies [SYNU], 24 with frontotemporal dementia [FTD] and 22 with idiopathic normal-pressure hydrocephalus [iNPH]) and 50 participants without neurodegenerative disorders (33 individuals undergoing knee arthroplasty and 17 with psychiatric diagnoses). pTau181 and pTau217 were measured via single-molecule array.

**Results:**

pTau181 differentiated AD patients from psychiatric patients with an area under the curve (AUC) of 0.879 and AD patients from all other participants with an AUC of 0.685. pTau181 was higher in patients with AD compared to FTD, iNPH, and non-neurodegenerative (ND) patients. pTau217 differentiated AD patients from psychiatric patients, with an AUC of 0.998, and AD patients from all other groups, with an AUC of 0.835. pTau217 was higher in AD patients compared to ND, FTD, and SYNU patients, but it did not differ between AD and iNPH patients without adjustment for age as a covariate.

**Conclusions:**

Our prospective cohort data indicate that pTau217 differentiates AD patients from psychiatric patients, with an excellent AUC value in receiver operating characteristic analysis. Our study supports the use of pTau217 rather than pTau181 as a minimally invasive tool to differentiate AD from non-neurodegenerative diseases (e.g., psychiatric disorders). Further studies are needed to determine the nature of pTau217 in iNPH.

## Introduction

Alzheimer's disease (AD) is a neurodegenerative disorder characterized by amyloid-β (Aβ) plaques and neurofibrillary tau deposits as abnormal findings in the brain parenchyma.^1^ In current clinical practice, the diagnosis requires the indication of biological hallmarks of AD, obtained via cerebrospinal fluid (CSF) biomarkers and amyloid positron emission tomography (PET) imaging,^1,2^ which are either costly, invasive or limited in terms of accessibility. Accessible, less invasive and economically sustainable methods are therefore urgently needed for diagnostic purposes.

Plasma-phosphorylated tau biomarkers have been suggested to serve as possible clinical diagnostic tools for AD.^3^ These biomarkers could most likely aid in differentiating AD from psychiatric disorders such as depression. This distinction is essential, since neuropsychiatric symptoms are prevalent in early stages of AD, including mild cognitive impairment.^4,5^ To our knowledge, no previous studies have examined the performance of pTau217 in patients with idiopathic normal-pressure hydrocephalus (iNPH) or psychiatric disorders. Previously, only two studies on plasma pTau181^6,7^ and two studies on plasma pTau217^7,8^ have addressed the ability of these biomarkers to differentiate AD from several other diagnostic groups of neurodegenerative diseases and controls**.** In this study, we utilize prospective cohort data regarding the performance of plasma pTau181 and pTau217 in differentiating AD from several other neurodegenerative disorders, psychiatric disorders and cognitively unimpaired controls. Participant groups in this study consist of participants with AD, synucleinopathies (SYNU), FTD, iNPH and non-neurodegenerative disorders. We selected these groups that reflect the diagnostic spectrum encountered in a real-world memory clinic. Furthermore, we evaluate these pTau isoforms with respect to neurodegenerative imaging findings.

## Methods

### Subjects

**Figure 1.**
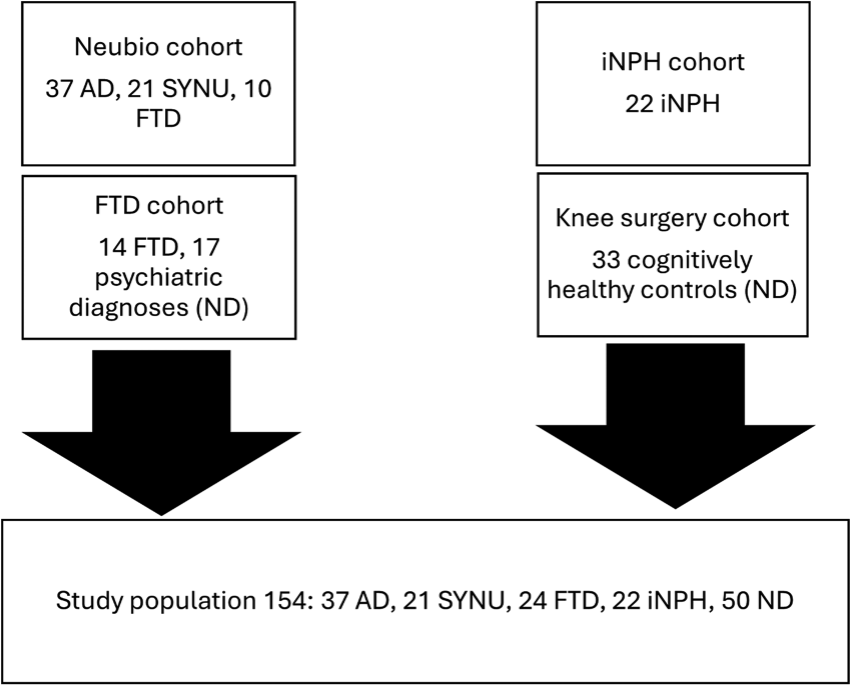
Flow chart illustrating the formation of the study population from cohorts for this study. Abbreviations of the group names are presented in Table 1.

The composition of the study population is presented in Figure 1. From four different cohorts (Kuopio Neubio cohort, iNPH cohort, frontotemporal dementia [FTD] cohort and knee surgery cohort), we identified 154 participants for this study. The Kuopio Neubio, iNPH and frontotemporal dementia cohorts include prospective sample collection and clinical variables for biomarker studies. The Kuopio Neubio cohort comprises patients with suspected neurodegenerative diseases. All patients with a neurodegenerative diagnosis or a psychiatric disorder were evaluated at Kuopio University Hospital. The study population consisted of patients with AD (N = 37), SYNU (N = 21), FTD (N = 24), iNPH (N = 22) and psychiatric disorders (N = 17) and cognitively unimpaired individuals undergoing knee surgery (N = 33). Patients with psychiatric diagnoses and patients undergoing knee surgery were included in the non-neurodegenerative group (ND, N = 50). Psychiatric patients were also assessed as a separate group in comparison with the AD group. Recruitment criteria for AD were at least two abnormal CSF markers among total tau, phosphorylated tau and amyloid-β 42 and a phenotype of either amnestic variant or posterior cortical atrophy. This recruitment protocol was able to yield an AD patient cohort fulfilling the Dubois criteria^2^ with the certainty level of at least possible AD. The SYNU group consisted of patients with Parkinson's disease^9^ (N = 13), Lewy body disease^10^ (N = 7) and multiple system atrophy^11^ (N = 1). Patients with neurodegenerative disorders fulfilled respective diagnostic criteria.^2,9–14^ For the patient group with psychiatric diagnoses, clinical judgement was used to exclude patients who were likely to have a concomitant neurodegenerative disorder. AD, SYNU, FTD and iNPH patients suspected to have another confounding neuropathology with related symptoms were excluded. Further details regarding participant groups can be found in the Supplemental Material.

**Table 1.** Demographic, biomarker and imaging data.

Category		Alzheimer's disease	Synucleinopathies	Frontotemporal dementia	Non-neurodegenerative (Psychiatric diagnoses and cognitively unimpaired)	Normal-pressure hydrocephalus	Total	p-values in statistical analysis
Abbreviation of group name		AD	SYNU	FTD	ND	iNPH		
Number of patients	N	37	21	pTau181 24 pTau217 23	50	22	pTau181 154 pTau217 153	
Age in years during blood sample collection	Median (Range)	66 (54–84)	67 (57–82)	64 (36–84)	69 (38–82)	73.5 (65–85)	68 (36–85)	<0.001
Number of females per group	N (%)	20 (54.1)	9 (42.9)	11 (45.8)	30 (60.0)	11 (50.0)	81 (52.6)	0.657
Mini-Mental State Examination (MMSE) score	Median (Range)	24 (11–30)	28 (14–30)	25 (14–29)	28 (23–30)	24 (19–29)	26 (11–30)	<0.001
MMSE result available	N (%)	35 (94.6)	17 (81.0)	19 (79.2)	46 (92.0)	22 (100.0)	139 (90.3)	0.067
pTau181 (pg/ml)	Median (Range)	3.71 (1.62–5.87)	3.17 (0.98–6.85)	2.26 (1.28–11.3)	2.34 (0.94–15.18)	2.05 (0.72–6.13)	2.69 (0.72–15.18)	0.007
pTau217 (pg/ml)	Median (Range)	0.67 (0.19–1.58)	0.35 (0.16–1.25)	0.20 (0.04–1.15)	0.28 (0.03–2.08)	0.44 (0.13–1.59)	0.35 (0.03–2.08)	<0.001
Age-adjusted median pTau217 level compared to AD (pg/ml)	Median (confidence interval; p-value compared to AD)	Comparator	−0.293 (−0.421 to −0.164; < 0.001)	−0.400 (−0.526 to −0.275; < 0.001)	−0.369 (−0.471 to −0.266; < 0.001)	−0.347 (−0478 to −0.216; <0.001)		p-values presented with age-adjusted medians
CSF markers available	N (%)	37 (100.0)	11 (52.4)	pTau181 15 (62.5) pTau217 14 (60.9)	37 (74.0)	22 (100.0)	pTau 181 122 (79.2) pTau217 121 (78.6)	<0.001
Brain MRI available for neurodegeneration evaluation (excluding NPH)	N (%)	34 (91.9)	14 (66.7)	24 (100.0)	13 (26.0)	Excluded	85 (63.3)	<0.001
Clinical Dementia Rating (CDR) distribution	N (%)	CDR 0 0 (0) CDR 0.5 26 (70.2) CDR 1 10 (27.0) CDR 2 1 (2.7)	CDR 0 10 (47.6) CDR 0.5 6 (28.6) CDR 1 4 (19.0) CDR 2 1 (4.8)	CDR 0 2 (8.3) CDR 0.5 9 (37.5) CDR 1 10 (41.7) CDR 2 3 (12.5)	CDR 0 42 (84.0) CDR 0.5 7 (14.0) CDR 1 1 (2.0) CDR 2 0 (0)	CDR 0 2 (9.1) CDR 0.5 17 (77.3) CDR 1 3 (13.6) CDR 2 0 (0)		<0.001

Patients with iNPH were significantly older compared to all other groups (p = 0.004; 0.007; < 0.001; and < 0.001 for AD, SYNU, FTD and ND participants, respectively). The MMSE scores of SYNU patients were significantly better compared to those of AD, FTD and iNPH patients (p = 0.014; 0.009; and 0.023, respectively). ND participants also exhibited significantly better MMSE scores compared to AD, FTD and iNPH patients (p < 0.001 in all comparisons). MRI data were available significantly more often for the AD and FTD groups (p < 0.001 for both) and significantly less often for the ND group (p < 0.001). CSF data were available significantly more frequently for AD patients (p < 0.001) but were available for significantly fewer SYNU patients (p = 0.001). Observed numbers of AD patients with a CDR score of 0, ND participants with CDR scores of 0.5 and 1 and FTD patients with a CDR score of 0 were lower than expected (p < 0.0025 for all). Observed numbers of AD patients with a CDR score of 0.5, ND participants with a CDR score of 0, iNPH patients with a CDR score of 0.5 and FTD patients with a CDR score of 1 were higher than expected (p < 0.0025 for all). In the AD group, only one patient displayed abnormal results of both CSF amyloid-β and total tau and a normal result of CSF phosphorylated tau. In the SYNU group, 19 patients of 21 were both CSF amyloid-β and CSF tau negative. Two patients in this group were CSF amyloid-β-positive and CSF-tau-negative. Continuous data (age and MMSE, pTau181 and pTau217 results) were analysed using the Kruskal–Wallis test. Categorical data (sex distribution within groups and the availability of MMSE results, brain imaging data and CSF marker data) were assessed using a chi-squared test and the method of adjusted residuals as a post hoc test.

Knee replacement surgery participants had completed the CERAD (Consortium to Establish a Registry for Alzheimer's Disease) assessment to rule out cognitive impairment. The Clinical Dementia Rating (CDR) was determined for cognitive stratification for all participants. Available clinical patient data, including data from the Mini-Mental State Examination (MMSE) and brain imaging, were obtained from electronic patient reports from Kuopio University Hospital. Neuropsychological testing data were assessed. Varying neuropsychological tests had been tailored by the treating neuropsychologist. Different subtests in neuropsychological testing included Trail Making, WAIS III subtests, Stroop, CERAD subtests including Naming and Clock Drawing. The magnetic resonance imaging (MRI) data consisted of Scheltens scored hippocampal atrophy data and Fazekas scored vascular degeneration data. The imaging data were evaluated by a clinician not blinded to other data. The acquisition of imaging data and the processing of available CSF data are described in the Supplemental Material.

### Frontal cortical biopsies of normal-pressure hydrocephalus patients

Most iNPH patients (20 out of 22 patients) had neuropathological data regarding tau and amyloid-β on a cortical biopsy sample which had been collected during a CSF shunting operation.^15,16^ Shunt surgery had been considered a necessary treatment for iNPH, and cortical biopsies were obtained during surgery, as described previously.^15^ Biopsy sample processing is explained in detail in the Supplemental Material.

### Plasma samples

Plasma phosphorylated tau isoforms pTau181 and pTau217 were measured with Simoa single-molecule array technology (Quanterix, Billerica, MA, USA). A Simoa HD-1 analyzer and pTau181 Advantage V2 kit (Quanterix #103714) were used for pTau181 analyses. pTau217 was analyzed with a Simoa HD-X analyzer using an ALZpath Simoa p-Tau217 v2 kit (Quanterix #104371) according to the manufacturer's instructions. In the standard procedure, the samples were diluted 1:4 by the analyzer. Due to the small sample volume, 22 pTau217 samples (from 8 FTD patients and 14 ND patients) were additionally prediluted 1:3 with sample diluent included in the kit prior to loading to the analyzer. This predilution was accounted for when assessing pTau217's statistical performance. We first used a Kruskal–Wallis test including these plasma samples and then reperformed the test excluding the prediluted samples. In the pTau217 analysis, the number of FTD patients was limited to 23 due to a lack of available plasma sample from one patient included in pTau181 analysis.

### Statistics

IBM SPSS version 29 (IBM Corporation, Armonk, NY, USA) was used for all statistical analyses. All graphs were generated using GraphPad Prism (GraphPad Software, Boston, MA, USA). Assessments of continuous variables between the diagnostic groups were performed using the Kruskal–Wallis test and its corresponding post hoc test. Comparisons of plasma pTau levels between sexes were performed using a Mann–Whitney U test. Correlations of continuous variables were analyzed using Spearman's rho. MMSE was considered a continuous variable. Differences in categorical data between groups were assessed using a chi-squared test and the method of adjusted residuals as a post hoc test. For this post hoc test, a Bonferroni correction was applied to adjust for multiple comparisons. Receiver operating characteristic (ROC) analyses, yielding area under the curve (AUC) values, were conducted to evaluate the diagnostic performance of both plasma pTau isoforms. Outlying pTau181 and pTau217 values were omitted from the Kruskal–Wallis test to confirm whether they affected the test results. Outlying pTau values were defined as those coinciding with interquartile range (IQR) values lower than quartile 1–1.5 IQR or higher than quartile 3 + 1.5 IQR. Generally, p-values ≤ 0.05 were considered significant. A Bonferroni correction determined significant p-values for multiple comparisons in a post hoc test. Differences between AUC values were tested with the DeLong test. Maximum values of Youden's index were determined to yield cut-off levels for both pTau181 and pTau217. Plasma pTau results of all participants in this study were used in these analyses to establish cut-off levels for AD as the target condition. These cut-off levels were used to calculate sensitivity, specificity, positive predictive value (PPV) and negative predictive value (NPV) in the entire study cohort. Sensitivity, specificity and predictive values were also calculated with established cut-off levels of pTau181 and pTau217 for differentiating the AD group from patients with psychiatric disorders. Median regression was performed to assess the role of age as a covariate with respect to pTau217 in different diagnostic groups. The role of cognitive status was assessed using median regression and CDR as a covariate with respect to pTau181 and pTau217 in different diagnostic groups. The AD group was used as the comparator group in these comparisons.

### Ethics statement

This study was conducted in accordance with the principles of the Declaration of Helsinki. The study protocol was approved by the ethics committee of the Northern Savo Hospital District (28.8.2013/42/2013). Participants provided written informed consent for this study.

## Results

### Patient characteristics

The demographic characteristics, biomarker and imaging data are presented in Table 1. The findings from the CSF analysis and cortical biopsy for iNPH patients are detailed in Table 2.

**Table 2.** CSF biomarker and biopsy data of iNPH patients. One iNPH patient with inconclusive CSF amyloid-β value is not shown to limit heterogeneity.

Category		Amyloid-β + Tau+	Amyloid-β + Tau-	Amyloid-β – Tau+	Amyloid-β –Tau-
iNPH CSF characteristics	N (%)	1 (4.5)	3 (13.6)	1 (4.5)	16 (72.7)
iNPH biopsy findings	N (%)	2 (9.1)	7 (31.8)	0 (0)	11 (50.0)

### Plasma pTau181 differentiates AD patients from FTD, iNPH, and ND patients

**Figure 2.**
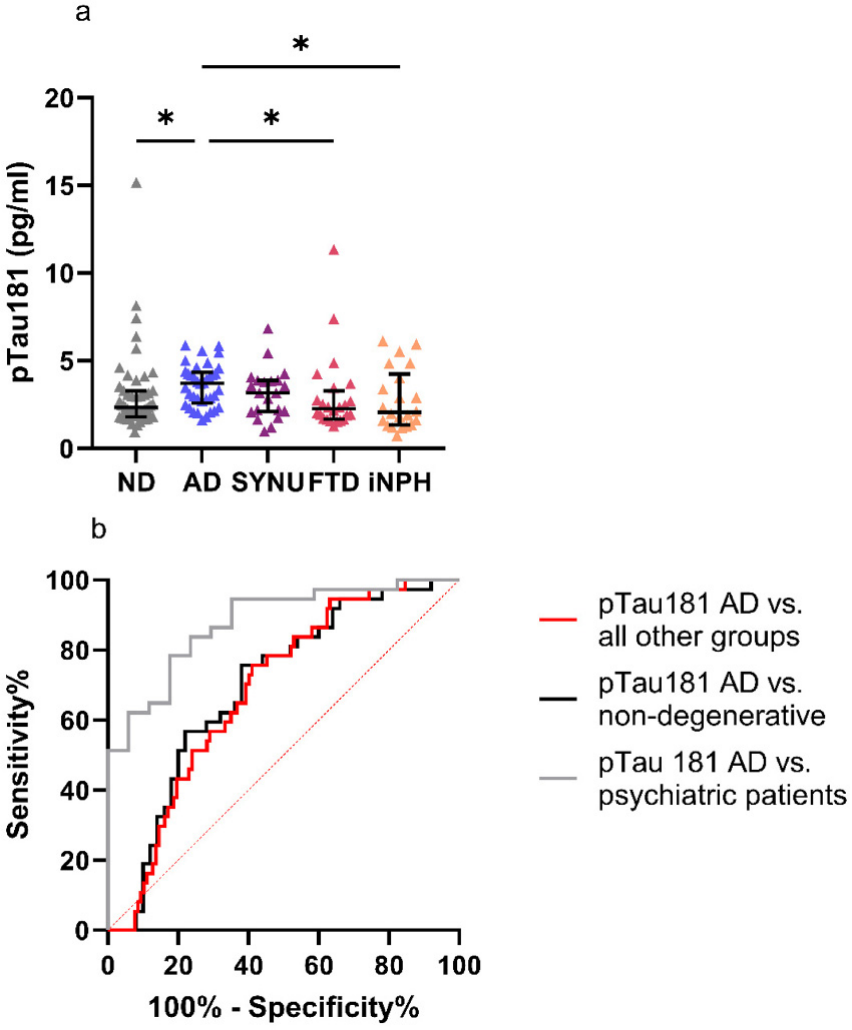
Plasma pTau181 (A) levels in different patient groups and ND participants and (B) in ROC analysis in separating AD patients from other groups. (A) AD patients displayed significantly higher plasma pTau181 levels compared to FTD patients, iNPH patients and ND participants with the Kruskal–Wallis test applied. (B) This figure illustrates the ability of plasma pTau181 in differentiating AD patients from all other groups (AUC 0.685), AD patients from all ND subjects (AUC 0.695) and AD patients from psychiatric patients (AUC 0.879). *p < 0.05.

Plasma pTau181 levels were significantly higher in patients with AD when compared to FTD patients, iNPH patients, and all ND participants (p = 0.034, 0.031, and 0.037, respectively). There were no significant differences between the pTau181 levels of AD patients and those of SYNU patients (Figure 2A). Eliminating five outlier values from the ND group, one from the SYNU group and two from the FTD group did not alter the results. The ROC analysis of pTau181 yielded AUC values of 0.879 (CI AUC 0.787–0.972), 0.695 (CI AUC 0.584–0.807), and 0.685 (CI AUC 0.597–0.774) when differentiating AD patients from psychiatric patients, from all ND participants and from all other diagnostic groups, respectively (Figure 2B). In median regression CDR as a covariate did not have a significant effect on pTau181 levels between the groups. pTau181 levels did not differ between sexes. The maximum Youden's index determined cut-off level for pTau181 was 2.71 pg/ml yielding a sensitivity of 0.76, a specificity of 0.59, an NPV of 0.89 and a PPV of 0.37 in the entire cohort. This cut-off level yielded a sensitivity of 0.76, a specificity of 0.82, an NPV of 0.61, and a PPV of 0.90 in differentiating the AD group from psychiatric patients.

### Plasma pTau217 can differentiate AD patients from FTD, SYNU and ND patients

**Figure 3.**
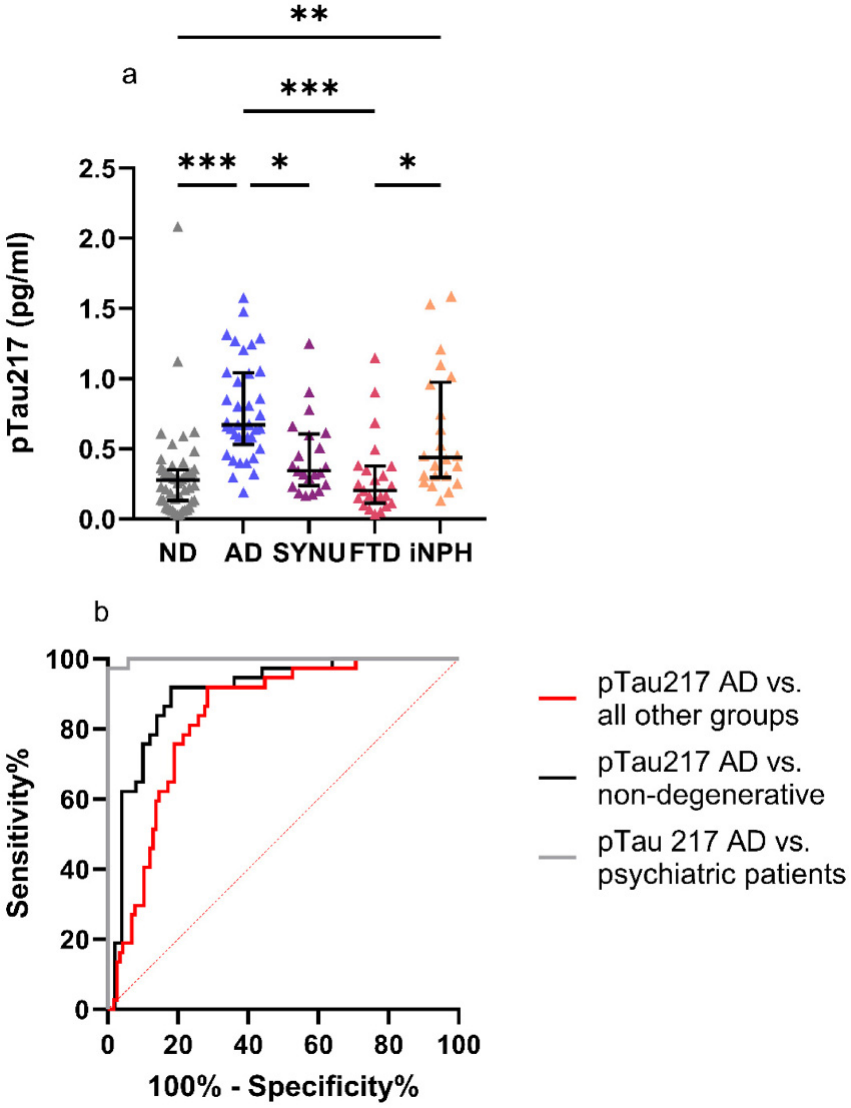
Plasma pTau217 (A) levels in different patient groups and ND participants and (B) in ROC analysis in differentiating AD patients from other groups. (A) Plasma pTau217 levels were significantly elevated in AD patients compared to SYNU patients, FTD patients and ND participants with the Kruskal–Wallis test applied. pTau217 levels were also elevated in iNPH patients compared to FTD patients and the ND group. (B) This figure illustrates the ability of plasma pTau217 in differentiating AD patients from all other groups (AUC 0.835), AD patients from all ND participants (AUC 0.902) and AD patients from psychiatric patients (AUC 0.998). ***p < 0.001, **p < 0.01, *p < 0.05.

AD patients exhibited significantly higher plasma pTau217 levels compared to the other patient groups aside from the iNPH patients (p < 0.001; < 0.001; = 0.022 for ND, FTD, and SYNU patients, respectively). iNPH patients exhibited significantly elevated pTau217 levels compared to FTD patients (p = 0.021) and ND subjects (p = 0.005) (Figure 3A). Eliminating outlier values (two in the ND group, one in the SYNU group and two in the FTD group) did not alter the results. We also tested whether eliminating the samples that had undergone the predilution process (described in the materials and methods sections) affected the results. Omitting the prediluted plasma samples did not alter the significant differences observed between AD patients’ pTau217 levels compared to those of SYNU, FTD, and ND subjects. With the prediluted plasma samples eliminated, the significant difference between the pTau217 levels in iNPH and FTD patients remained (p = 0.048). However, this elimination led to the disappearance of the significant difference between the pTau217 levels in the iNPH and ND groups (p = 0.234). The median regression demonstrated a significant difference between age-adjusted median pTau217 levels of AD patients compared to all other diagnostic groups (all p < 0.001) (Table 1). An ROC analysis of pTau217 yielded AUC values of 0.998 (CI AUC 0.993–1.000), 0.902 (CI AUC 0.835–0.970) and 0.835 (CI AUC 0.768–0.901) when differentiating AD patients from psychiatric patients, all ND subjects and all other groups in this study, respectively (Figure 3B). CDR as a covariate had no significant effect on pTau217 levels between the groups in the median regression analysis. pTau217 levels did not differ between sexes. All AUC values of pTau217 were significantly higher compared to AUC values of pTau181 in the DeLong test (p = 0.012; <0.001; <0.001 when comparing the differentiation between AD patients and psychiatric patients, AD patients and all ND participants and AD patients and all other groups, respectively). The maximum Youden's index determined cut-off level for pTau217 was 0.39 pg/ml yielding a sensitivity of 0.92, a specificity of 0.72, an NPV of 0.97 and a PPV of 0.51 in the entire cohort. This cut-off yielded a sensitivity of 0.92, a specificity of 1, an NPV of 0.85 and a PPV of 1 in differentiating the AD group from psychiatric patients.

### Correlations of biomarkers and patient characteristics

**Figure 4.**
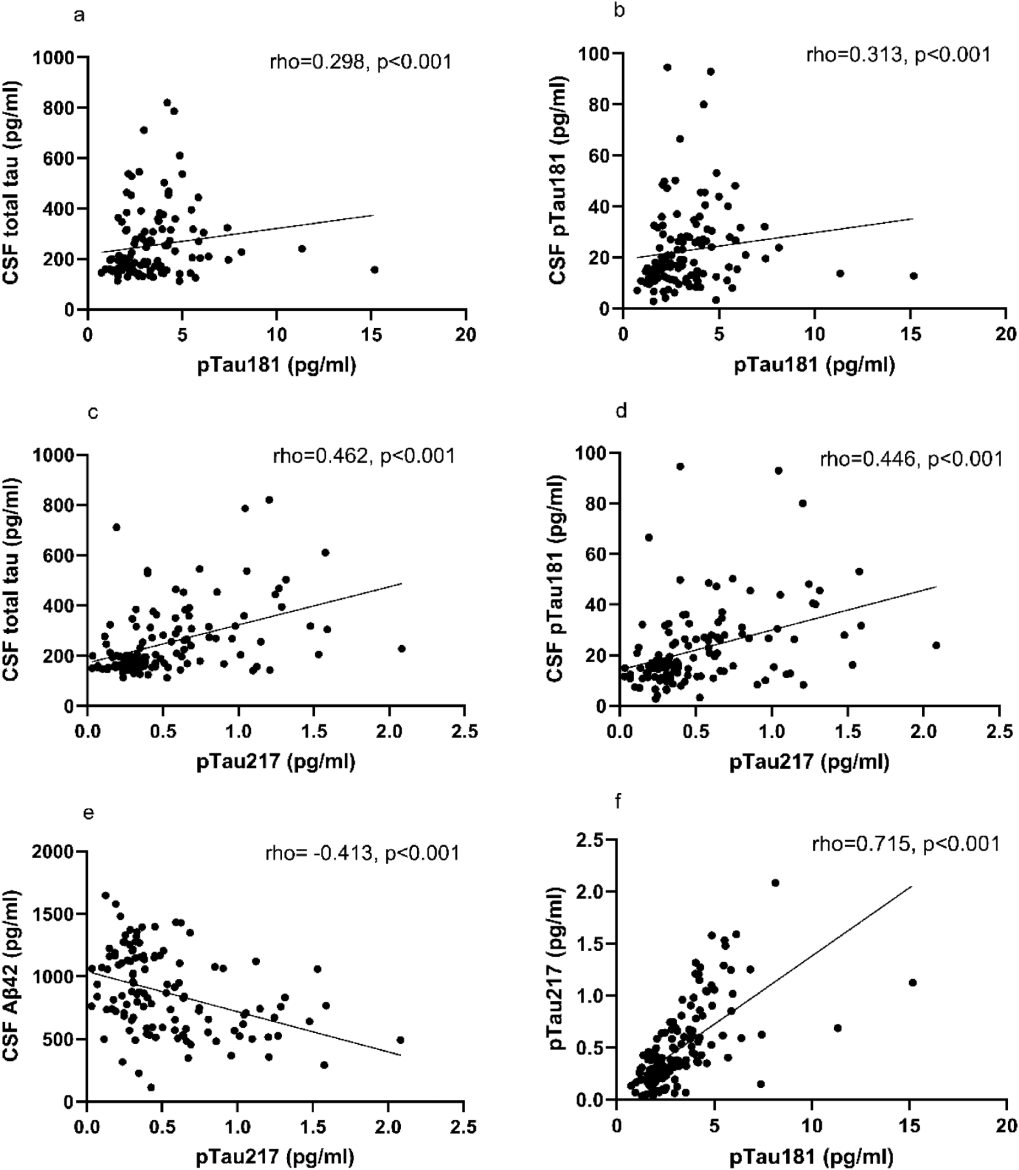
Spearman's correlations of plasma pTau181 levels with CSF total and phosphorylated tau and of plasma pTau217 levels with all CSF biomarkers and between the plasma pTau isoforms. (A, B) The correlations of plasma pTau181 with CSF total tau and CSF pTau181. (C, D, E) The correlations of plasma pTau217 with CSF total tau, CSF pTau181 and CSF amyloid-β 42. (F) The correlation of plasma pTau217 with plasma pTau181.

Correlations of plasma pTau isoforms with CSF biomarkers and with each other are illustrated in Figure 4. There was a significant correlation between plasma pTau181 and CSF total tau levels (r_s_ = 0.298, p < 0.001) and between plasma pTau181 and CSF pTau181 levels (r_s_ = 0.313, p < 0.001). CSF amyloid-β 42 (Aβ_42_) and plasma pTau181 levels did not exhibit a significant correlation (r_s_ = −0.123, p = 0.177). However, plasma pTau217 levels showed a significant negative correlation with CSF Aβ_42_ levels (r_s_ = −0.413, p < 0.001). Significant correlations also existed between plasma pTau217 and CSF total tau levels (r_s_ = 0.462, p < 0.001) and CSF pTau181 levels (r_s_ = 0.446, p < 0.001). Both plasma pTau isoform levels strongly correlated with each other (r_s_ = 0.715, p < 0.001). MMSE results and plasma pTau181 levels did not exhibit a statistically significant correlation. However, plasma pTau217 levels and MMSE scores significantly correlated with each other (r_s_ = −0.224, p = 0.008). The age of the patients at the time of the sample collection correlated with both plasma pTau181 and pTau217 levels (r_s_ = 0.282, p < 0.001 and r_s_ = 0.391, p < 0.001, respectively).

### Plasma pTau217 and pTau181 differentiating CSF biomarker profiles

**Figure 5.**
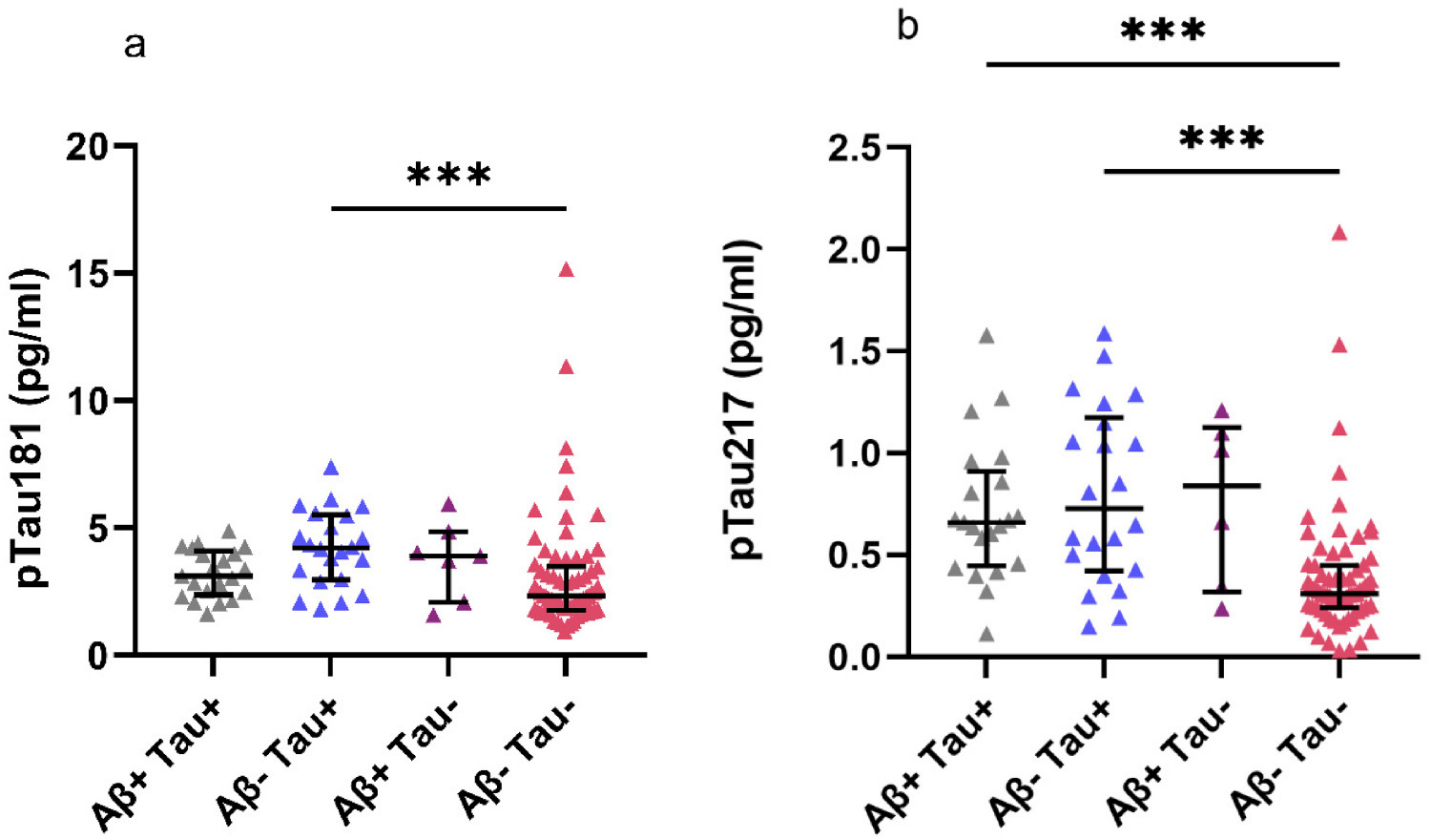
Plasma pTau181 and pTau217 levels with respect to different CSF marker profiles. (A) Plasma pTau181 levels differed significantly with respect to CSF marker profiles in the Kruskal–Wallis test. (B) Plasma pTau217 levels also differed with respect to CSF marker profiles in the Kruskal–Wallis test. Significant differences between groups are displayed in both figures. ***p ≤ 0.001.

Plasma pTau181 was significantly higher in CSF amyloid-β-negative and tau-positive patients (mean pTau181 4.20) compared to patients who were negative for both CSF biomarkers (mean pTau181 3.06, p = 0.001). Other groupwise comparisons of pTau181 levels with respect to different CSF biomarker profiles indicated no statistically significant differences (Figure 5A). pTau217 levels were significantly higher in patients exhibiting CSF tau positivity and amyloid-β positivity (mean pTau217 0.712, p < 0.001) and in patients who were CSF tau positive and amyloid-β negative (mean pTau217 0.797, p < 0.001) when compared to patients who were negative for both CSF biomarkers (mean pTau217 0.389). Other groupwise comparisons of pTau217 levels with respect to CSF biomarker profiles indicated no statistically significant differences (Figure 5B).

### Plasma pTau217 and pTau181 differentiating iNPH biopsy histological profiles

Comparing plasma pTau181 values in iNPH patients divided into separate subcategories based on their amyloid-β and tau status in the cortical biopsy samples revealed significant differences between these subcategories (p = 0.042), with tau-negative and amyloid-β-negative patients exhibiting the lowest pTau181 levels (mean pTau181 1.93) and amyloid-β- and tau-positive patients displaying the highest pTau181 levels (mean pTau181 4.21). However, these observed differences in pTau181 levels with respect to neuropathological groups were statistically insignificant in a post hoc test. Similarly, pTau217 levels differed in iNPH patients of different amyloid-β and tau status (p = 0.022), with tau- and amyloid-β-negative patients demonstrating the lowest pTau217 levels (mean pTau217 0.340) and tau- and amyloid-β-positive patients exhibiting the highest pTau217 levels (mean pTau217 1.139). Again, however, these observed differences were statistically insignificant in the Kruskal–Wallis post hoc test.

### Plasma pTau181 and pTau217 differentiating imaging findings of neurodegeneration

Comparing patients’ hippocampal atrophy grades and pTau181 results, the Kruskal–Wallis test revealed a significant difference between groups (p = 0.032), with participants of grade 0 showing the lowest pTau181 levels (mean pTau181 2.46) and participants of grade 3 exhibiting the highest pTau181 levels (mean pTau181 4.75). However, the post hoc analysis failed to uncover any groupwise differences. Likewise, pTau217 levels differed significantly when comparing grades of hippocampal atrophy (p = 0.010), with participants of grade 0 exhibiting the lowest pTau217 levels (mean pTau217 0.347) and participants of grade 2 displaying the highest pTau217 levels (mean pTau217 0.709). This difference between the two groups was also statistically significant in the post hoc test (p = 0.011). All other differences between groups were statistically insignificant. A comparison of different levels of brain vascular degeneration in relation to either pTau181 or pTau217 levels showed no significant differences between the groups.

## Discussion

We demonstrated that plasma pTau217 was able to differentiate AD patients from patients with various other types of neurodegenerative disease and specifically from those with psychiatric disorders**.** pTau217 was unable to distinguish AD patients from iNPH patients without adjustment for age as a covariate. We also revealed that plasma pTau217 correlates with the AD CSF biomarkers total tau, pTau181 and amyloid-β. We also found both plasma pTau isoforms to correlate with age.

To our knowledge, we report here for the first time results of plasma pTau217 differentiating AD patients from patients with psychiatric disorders. A very recent publication has also detailed a similar finding of pTau217 differentiating AD patients from patients with psychiatric disorders robustly.^17^ Our findings suggest that plasma pTau217 may be a useful biomarker in clinical settings when differentiating AD patients from patients with psychiatric disorders. This distinction is essential, as neuropsychiatric symptoms are prevalent in early stages of AD.^4,5^ Furthermore, to our knowledge, this study is the first to assess plasma pTau217 levels in iNPH patients. Our results demonstrate that pTau217 was not able to differentiate AD patients from iNPH patients without adjusting for age as a covariate. An age-adjusted model was able to uncover a significant difference between pTau217 levels of the AD group and the iNPH group. iNPH patients are known to often have co-occurring AD-like brain pathology.^18^ iNPH patients are also at a greater risk of developing AD compared to the general population. Further studies are essential in determining whether plasma pTau217 reflects comorbid AD pathology in iNPH. Most of the previous publications^7,8,19–27^ on pTau217 did not draw comparisons between multiple different neurodegenerative diseases, a dimension we found important to evaluate. Our findings are consistent with previous studies indicating the differential diagnostic capability of pTau217 in a study population of patients with multiple different neurodegenerative disorders and non-neurodegenerative participants.^7,8^ The AUC value was acceptable when evaluating the performance of pTau217 in the diagnostic differentiation of AD from all other diagnostic groups in the entire cohort. The NPV of 0.97 for pTau217 was comparable and even higher than the NPV for predicting amyloid-β in a recent publication.^28^ Our observed PPV of 0.51 for pTau217 was lower compared to PPVs recently published by others.^28^ However, this might be explained by differences in study population composition, and we are unaware of other publications of pTau217 with multiple different neurodegenerative groups providing test predictive values. Our observed high NPV and low PPV for the entire study cohort suggest that pTau217 might have value as a diagnostic method to rule AD out in a memory clinic setting. Given the low observed PPV of pTau217, abnormal results for pTau217 should entail confirmatory testing in a memory clinic setting before AD is diagnosed. The role of cognitive status assessed by CDR had no significant effect on the results for pTau217 between the groups. Most patients with neurodegenerative disorders in this study had been assessed in an early phase of the disorder.

According to previous studies, elevated plasma pTau217 levels indicate both amyloid-β and tau pathologies in the brain.^19,20^ In our study, plasma pTau217 correlated positively with CSF total tau and pTau and negatively with CSF amyloid-β. Similar correlations have been reported for CSF pTau^7,8^ and for CSF amyloid-β.^21^ Furthermore, in our study, plasma pTau217 levels were higher, with patients exhibiting abnormal CSF profiles (amyloid positive and tau positive or amyloid negative and tau positive). This study corroborates the previous findings suggesting that pTau217 is a marker of both amyloid-β and tau pathologies.^19,20^ The small group size of iNPH patients likely contributed to the analysis results of different brain biopsy categories, and the analysis thus did not reveal significant differences of plasma pTau isoform levels between different categories of neuropathology findings with respect to amyloid-β and tau.

According to earlier publications, pTau181 correlates negatively with medial temporal lobe volume^29,30^ and specifically hippocampal volume in imaging.^6,31–33^ pTau217 has previously been demonstrated to correlate negatively with hippocampal volume,^33^ and another study indicated that both plasma pTau181 and pTau217 correlate negatively with hippocampus subregion volume in imaging.^34^ Hippocampal atrophy in imaging is recognized as a typical feature in AD.^35^ We were able to uncover a significant difference between the pTau217 levels of Scheltens 2 and Scheltens 0 categories via MRI. Our study may have been underpowered to demonstrate significant groupwise differences of pTau181 levels with respect to different hippocampal atrophy grades. In this study, comparisons of plasma pTau isoforms and neurodegenerative findings were completed using imaging results evaluated by a clinician. This might have affected our results, as most studies use volumetric analysis to evaluate brain atrophy. We opted to use a clinician's opinion for imaging data, as many radiology reports lacked numerical gradings of hippocampal atrophy and vascular degeneration. The lack of correlation between vascular degeneration and plasma pTau isoform levels can most likely be explained by ischemia as the etiology behind vascular degeneration,^36^ as opposed to amyloid and tau pathology.

In our study, plasma pTau217 levels correlated with MMSE scores, as demonstrated earlier.^7^ CSF and PET imaging, acting as biomarkers of tau pathology, correlate with patients’ cognitive decline.^37,38^ As pTau217 has previously been suggested as a marker of brain tau pathology,^19,20^ elevated plasma pTau217 levels may indicate a risk of cognitive decline linked to brain tau pathology. An earlier publication has asserted that CSF markers of tau pathology may act as prognostic factors of cognitive decline.^37^ Another publication focusing on both amyloid and tau PET postulated that cognitive decline in AD is linked to tau pathology, as opposed to amyloid pathology.^38^ Neuropathological studies have corroborated these findings of cognitive impairment correlating with the neocortical accumulation of pathological tau.^39^ This association of plasma pTau217 with brain tau pathology may explain the negative correlation between pTau217 and MMSE. We were also able to reproduce a previous finding wherein pTau217 and pTau181 correlated strongly with each other.^7,21,23,26^ Plasma pTau217 levels also correlated with advanced age, a finding reported in a previous study.^23^ Amyloid positivity was observed as a factor contributing to this correlation between pTau217 levels and advanced age. However, a positive correlation has also been observed for the amyloid-negative study subjects.^23^ In the future, this correlation should be considered when interpreting the results regarding pTau217 amongst patients of different ages.

In this study, plasma pTau181 levels were also able to differentiate AD patients from all ND subjects, FTD patients and iNPH patients. However, plasma pTau181 failed to differentiate AD patients from SYNU patients. Furthermore, all AUC values of pTau181 for the differentiation of AD from the other diagnostic categories were significantly lower than those of pTau217. The lower AUC values observed do not encourage the clinical use of pTau181 as a biomarker in differential diagnostics of AD. Our study confirmed the already-reported results indicating that plasma pTau181 levels are elevated with advanced age^23,40,41^ and that pTau181 levels are correlated with CSF pTau levels.^6,42^

Our study also lends support to the notion that pTau217 is a useful biomarker differentiating AD patients from patients with other diagnoses, particularly non-degenerative etiologies (e.g., psychiatric disorders). In ROC analysis, the AUC value for pTau217 appeared excellent when comparing AD patients and psychiatric patients. Previously, plasma pTau217 has been considered suitable for clinical implementation studies,^24^ and our findings corroborate this opinion.

### Limitations

Here, we report for the first time the results of plasma pTau217 levels in iNPH patients. Further studies are essential to determine the nature of pTau217 in relation to iNPH. Moreover, future studies are required to determine whether elevated pTau217 reflects comorbid AD pathology in iNPH. pTau217 performed especially robustly in differentiating AD patients from patients with psychiatric disorders. Similar results indicating excellent performance of pTau217 differentiating AD from psychiatric disorders have been published recently.^17^ We recognize that the number of participants in the study groups was restricted, which might have influenced the results. This limitation in group sizes was addressed in another publication regarding pTau217,^8^ and, as stated before, groupwise comparisons should be interpreted with caution due to this limitation. Our data suggest that singular patients can have remarkably high levels of plasma pTau isoforms irrespective of their diagnostic group. Understandably, this phenomenon must be considered when interpreting individuals’ results. Our study design lacked confirmation of the diagnosis of a neurodegenerative disorder in the autopsy. As a result, patients might have harbored multiple concomitant degenerative brain pathologies despite the stringent inclusion criteria of the specific diagnostic categories. However, according to our observations, this limitation of lacking autopsy confirmation applies to most published studies in the field.^19–27^ Previous autopsy studies have noted that aged people may carry multiple neurodegenerative pathologies simultaneously.^43,44^ This phenomenon might have affected our results, as the AUC value of plasma pTau217 was lower in differentiating AD patients from all other diagnostic categories in this study. Some limitations exist concerning cortical biopsy. Right frontal cortex biopsy is a highly sensitive method to detect amyloid-β pathology when compared to either amyloid PET or postmortem neuropathological examination.^45,46^ However, patchy amyloid-β pathology can still be missed in cortical biopsy. For tau pathology the cortical biopsy has a high positive predictive value, yet sensitivity is low in the early stages of AD. The low sensitivity is explained by cortical tau appearance only in the later stages (Braak V-VI) in AD.^47^ Frontal cortical biopsy was used as a proxy marker for AD pathology, as opposed to establishing a neuropathological AD diagnosis. In the SYNU, FTD and ND groups, CSF biomarkers were not available for all participants. This reflects the real-world clinical practice diagnostic approach to FTD, synucleinopathies and psychiatric disorders. The incomplete availability of CSF biomarkers in these groups is also in accordance with the ethical approval for this study as CSF collection had to be clinically indicated in these groups. Consistently available neuropsychological data were limited to MMSE and CDR in this study as no uniform neuropsychological testing was performed simultaneously with blood sample collection.

### Conclusion

In this study, we provide novel findings of the robust capability of plasma pTau217 in differentiating AD from a comparison group with psychiatric disorders in a real-world study population of a memory clinic. We also provide novel results of pTau217 in iNPH cohort with accompanying cortical biopsy data. Our results demonstrate that pTau217 differentiates AD from various other neurodegenerative disorders, psychiatric disorders and cognitively unimpaired controls. Further studies would help determine whether plasma pTau217 measurements may prove useful in the future in primary care in differentiating AD patients from patients with other diagnoses (e.g., those with psychiatric disorders). The enrollment of patients with iNPH in further studies is also necessary to determine the nature of pTau217 in iNPH. Further studies of pTau217 with various diagnostic groups and additional blood-based biomarkers, such as glial fibrillary acid protein and neurofilament light chain, are also called for to provide complementary information about the mechanisms of neurodegenerative disorders.

## Supplemental Material

sj-docx-1-alz-10.1177_13872877261431800 - Supplemental material for The performance of plasma pTau181 and pTau217 in distinguishing Alzheimer's disease from various neurodegenerative disorders, psychiatric disorders, and cognitively unimpaired controls Supplemental material, sj-docx-1-alz-10.1177_13872877261431800 for The performance of plasma pTau181 and pTau217 in distinguishing Alzheimer's disease from various neurodegenerative disorders, psychiatric disorders, and cognitively unimpaired controls by Juho-Antti Rissanen, Sari Kärkkäinen, Kasper Katisko, Aleksi Vanninen, Antti J. Luikku, Tuomas Rauramaa, Tadeusz Musialowicz, Merja Kokki, Valtteri Julkunen, Anne M. Portaankorva, Annakaisa Haapasalo, Eino Solje, Päivi Hartikainen, Ville Leinonen, Tarja Kokkola and Sanna-Kaisa Herukka in Journal of Alzheimer's Disease
