# Supplementary material for: The performance of plasma pTau181 and pTau217 in distinguishing Alzheimer's disease from various neurodegenerative disorders, psychiatric disorders, and cognitively unimpaired controls
Source: J Alzheimers Dis. 2026 Mar 23;111(1):385–96. doi: 10.1177/13872877261431800 (PMC13110333; doi:10.1177/13872877261431800)
Supplement: sj-docx-1-alz-10.1177_13872877261431800 - Supplemental material for The performance of plasma pTau181 and pTau217 in distinguishing Alzheimer's disease from various neurodegenerative disorders, psychiatric disorders, and cognitively unimpaired controls [file sj-docx-1-alz-10.1177_13872877261431800.docx]

**Supplemental Material**

**The performance of plasma pTau181 and pTau217 in distinguishing Alzheimer’s disease from various neurodegenerative disorders, psychiatric disorders, and cognitively unimpaired controls**

**Subjects**

Recruitment criteria for AD were at least two abnormal CSF markers among total tau, phosphorylated tau and amyloid-β 42 and a phenotype of either amnestic variant or posterior cortical atrophy. AD patients with a clinically reasonable suspicion of any other confounding neuropathology were excluded. For example, a patient with electronic records reporting having either AD or Lewy body disease (LBD) could not be included in either AD group or SYNU group. Any such patients were excluded. This recruitment protocol was able to yield an AD patient cohort fulfilling the Dubois criteria^1^ with the certainty level of at least possible AD**.** In the SYNU group, the PD patients fulfilled the certainty level of at least clinically probable PD.^2^ LBD patients fulfilled diagnostic criteria^3^ with a certainty level of at least possible. The MSA patient fulfilled the MDS 2022^4^ criteria with a diagnostic certainty level of clinically probable. FTD patients were required to fulfil either the criteria of Gorno-Tempini for linguistic variants (five patients) or Rascovsky for the behavioral variant FTD (19 patients) with a diagnostic level of at least probable for Rascovsky and clinical diagnosis for Gorno-Tempini.^5,6^ iNPH patients showed symptoms including decreased gait velocity, urinary incontinence and cognitive impairment^7^ and were classified as probable iNPH based on Relkin’s 2005 classification criteria.^8^ The iNPH group were part of the Kuopio iNPH cohort and had undergone a diagnostic and prognostic workup for iNPH.^9^ All patients had undergone CSF shunt surgery for iNPH according to the Kuopio iNPH protocol.^9^ Psychiatric diagnoses consisted of bipolar disorder, schizophrenia, depression, delusional disorder, eating disorder, generalized anxiety disorder, panic disorder, personality disorders (obsessive-compulsive personality, borderline personality, schizotypal personality).

**Imaging data acquisition**

Imaging results of patients within groups of AD, SYNU, FTD, and patients with psychiatric diagnoses in the control group were evaluated with a focus on brain magnetic resonance imaging (MRI) findings. The imaging results of patients with iNPH were evaluated to ensure the fulfilment of diagnostic criteria. When MRI imaging results were not available, computed tomography (CT) imaging results were accepted for evaluation of the fulfilment of Relkin’s diagnostic criteria in the iNPH group. Grades of both hippocampal atrophy and vascular degeneration were analyzed from patient records of patients with AD, SYNU, FTD and psychiatric diagnoses in the ND group. Hippocampal atrophy was assessed using Scheltens scoring and vascular degeneration evaluated using Fazekas scoring. MRI imaging was acquired using 1.5T or 3T scanners from Kuopio University Hospital. Scanners, both MRI and CT for iNPH group, were those used for diagnostic purposes in the Radiology Department of Kuopio University Hospital. Imaging using multiple scanners had been executed, as multiple scanners were available for diagnostic imaging. Imaging results were evaluated by a clinician, and data from radiology reports were obtained. The clinician compared MRI images with a respective report produced by the radiologist. When a report omitted assessment of hippocampal atrophy or vascular degeneration, the clinician conducted a substituting evaluation. This substituting evaluation was performed for 15 cases (17.6% of imaging data). In a case of a mismatch between a radiology report and an imaging evaluation performed by a clinician, a clinician’s opinion concerning degenerative findings was used for the data. This mismatch occurred in only three cases (3.5% imaging data). The clinician had five years of clinical experience in neurology and was not blinded to other patient data. In the case of a patient representing dissimilar grades of hippocampal atrophy between temporal lobes (e.g., left temporal lobe representing Scheltens grade 2 degeneration and the right representing Scheltens grade 1 degeneration), the higher value was selected in the data collection. Hippocampal atrophy was assessed mainly using coronal T1 sequences and in two cases with missing T1 coronal sequences with coronal T2 sequences. For data collection, imaging results of the primary diagnostic phase of neurological or psychiatric evaluation were used. MRI imaging occurred in a similar diagnostic phase with the blood sample collection, apart from five PD patients in the SYNU group. These five patients had undergone imaging in an earlier phase of the disease years before the blood sample collection. Two other SYNU patients with LBD had suitable data for vascular degeneration from the primary diagnostic phase but lacked data on hippocampal atrophy. Vascular degeneration data for these patients were included in the data set. To avoid bias, iNPH patients were not included in the data collection of neurodegenerative imaging findings. iNPH can lead to the misinterpretation of these imaging findings, as iNPH can exhibit periventricular hypodensities and lateral ventricle temporal horn dilation,^10^ which can be erroneously interpreted as vascular degeneration and hippocampal atrophy, respectively.

**CSF samples**

If CSF sample data from the diagnostic phase were available from patient records, these data were included. As mentioned before, abnormal CSF results for total tau, pTau181 and amyloid-β 42 were part of the inclusion criteria of AD patients in this study. Comparisons of the CSF marker profile and plasma pTau isoform levels were performed. In this CSF marker profiling, patients were divided into four separate groups based on tau and amyloid-β status (e.g., tau-negative, amyloid-β negative). Patients showing amyloid-β results in the grey zone (580–635 pg/ml using Innotest, 2 participants) were excluded from this comparison to eliminate heterogeneity and to limit the number of analyzed groups. CSF tau-positive participants were defined as participants displaying either at least positive CSF total tau or CSF pTau. All tau-positive participants exhibited at least either positive CSF total tau or CSF pTau results. No participants exhibiting both CSF tau biomarkers in the grey zone were encountered. Of all 43 CSF tau-positive participants, 38 participants showed abnormal results for both CSF total tau and CSF pTau (88.4% of all CSF tau-positive participants). Of all CSF samples (N = 122), 106 (86.9%) were obtained during the same day with the blood sample. In four participants (3.3%) the collection of CSF and blood samples had occurred less than six months apart, in three participants (2.5%) between six and 12 months apart, in four participants (3.3%) between one and two years apart. In one participant (0.8%) sample collection occurred less than five years apart. For one participant data of CSF collection date were missing.

CSF sample analyses had been carried out using a commercial enzyme-linked immunosorbent assay (Innostest, Fujirebio, Ghent, Belgium) before the beginning of 2020. Starting from January 2020, CSF sample assays were carried out using automated Elecsys immunoassays (Roche Diagnostics, Penzberg, Germany). Innotest results were converted to values comparable to Elecsys levels using equations presented in a previous study.^11^ These equations have been established previously in the University of Eastern Finland Biomarker Laboratory assessing 100 CSF samples using both methods mentioned above.

**Frontal cortical biopsies of normal-pressure hydrocephalus patients**

Frontal cortical biopsies were obtained during CSF shunt surgeries according to the Kuopio NPH registry study protocol, as described previously.^9^ The paraffin-embedded biopsy samples were sectioned (7 µm) and stained with hematoxylin-eosin and immunostained with monoclonal antibodies directed to amyloid-β (Aβ) (6F/3D, M0872; Dako; dilution 1:100; pretreatment 80% formic acid 1 hour) and hyperphosphorylated tau (HPτ) (AT8, 3Br-3; Innogenetics; dilution 1:30), as described previously.^12^ In all samples, immunoreactivity for Aβ and HPτ was graded as present or absent by a neuropathologist.^13^ In total, cortical brain biopsy data was available for 20 patients, with two cases of missing histological data.

**References**

1. Dubois B, Villain N, Frisoni GB, et al. Clinical diagnosis of Alzheimer’s disease: recommendations of the International Working Group. *Lancet Neurol* 2021; 20: 484–96.

2. Postuma RB, Berg D, Stern M, et al. MDS clinical diagnostic criteria for Parkinson’s disease. *Mov Disord* 2015; 30: 1591–1599.

3. McKeith IG, Boeve BF, Dickson DW, et al. Diagnosis and management of dementia with Lewy bodies Fourth consensus report of the DLB Consortium. *Neurology* 2017; 89: 88–100.

4. Wenning GK, Stankovic I, Vignatelli L, et al. The Movement Disorder Society Criteria for the diagnosis of multiple system atrophy. *Mov Disord* 2022; 37: 1131–1148.

5. Gorno-Tempini ML, Hillis AE, Weintraub S, et al. Classification of primary progressive aphasia and its variants. *Neurology* 2011; 76: 1006–1014.

6. Rascovsky K, Hodges JR, Knopman D, et al. Sensitivity of revised diagnostic criteria for the behavioral variant of frontotemporal dementia. *Brain* 2011; 134: 2456–2477.

7. Williams MA, Nagel SJ, Luciano MG, et al. The clinical spectrum of hydrocephalus in adults: Report of the first 517 patients of the Adult Hydrocephalus Clinical Research Network Registry. *J Neurosurg* 2020; 132: 1773–1784.

8. Relkin N, Marmarou A, Klinge P, et al. Diagnosing idiopathic normal-pressure hydrocephalus. *Neurosurgery* 2005; 57: S4-16.

9. Junkkari A, Luikku AJ, Danner N, et al. The Kuopio idiopathic normal pressure hydrocephalus protocol: initial outcome of 175 patients. *Fluids Barriers CNS* 2019; 16: 1–12.

10. Kockum K, Lilja-Lund O, Larsson E-M, et al. The idiopathic normal-pressure hydrocephalus Radscale: a radiological scale for structured evaluation. *Eur J Neurol* 2018; 25: 569–576.

11. Vanninen A, Lukkarinen H, Kokkola T, et al. Cerebrospinal fluid diagnostics of Alzheimer’s disease in patients with idiopathic normal pressure hydrocephalus. *J Alzheimers Dis* 2023; 94: 727–736.

12. Leinonen V, Koivisto AM, Savolainen S, et al. Post-mortem findings in 10 patients with presumed normal-pressure hydrocephalus and review of the literature. *Neuropathol Appl Neurobiol* 2012; 38: 72–86.

13. Alafuzoff I, Pikkarainen M, Arzberger T, et al. Inter-laboratory comparison of neuropathological assessments of β-amyloid protein: A study of the BrainNet Europe consortium. *Acta Neuropathol* 2008; 115: 533–546.
